# Supplementary material for: Thermal Stability and Crystallization Processes of Pd78Au4Si18 Thin Films Visualized via In Situ TEM
Source: Nanomaterials (Basel). 2024 Apr 5;14(7):635. doi: 10.3390/nano14070635 (PMC11013854; doi:10.3390/nano14070635)
Supplement: Supplementary file 1 [file nanomaterials-14-00635-s001.zip › Supplementary figures.pdf]

Supplementary Materials

Thermal Stability and Crystallization Processes of $Pd_{78}Au_4Si_{18}$ Thin Films Visualized via In Situ TEM

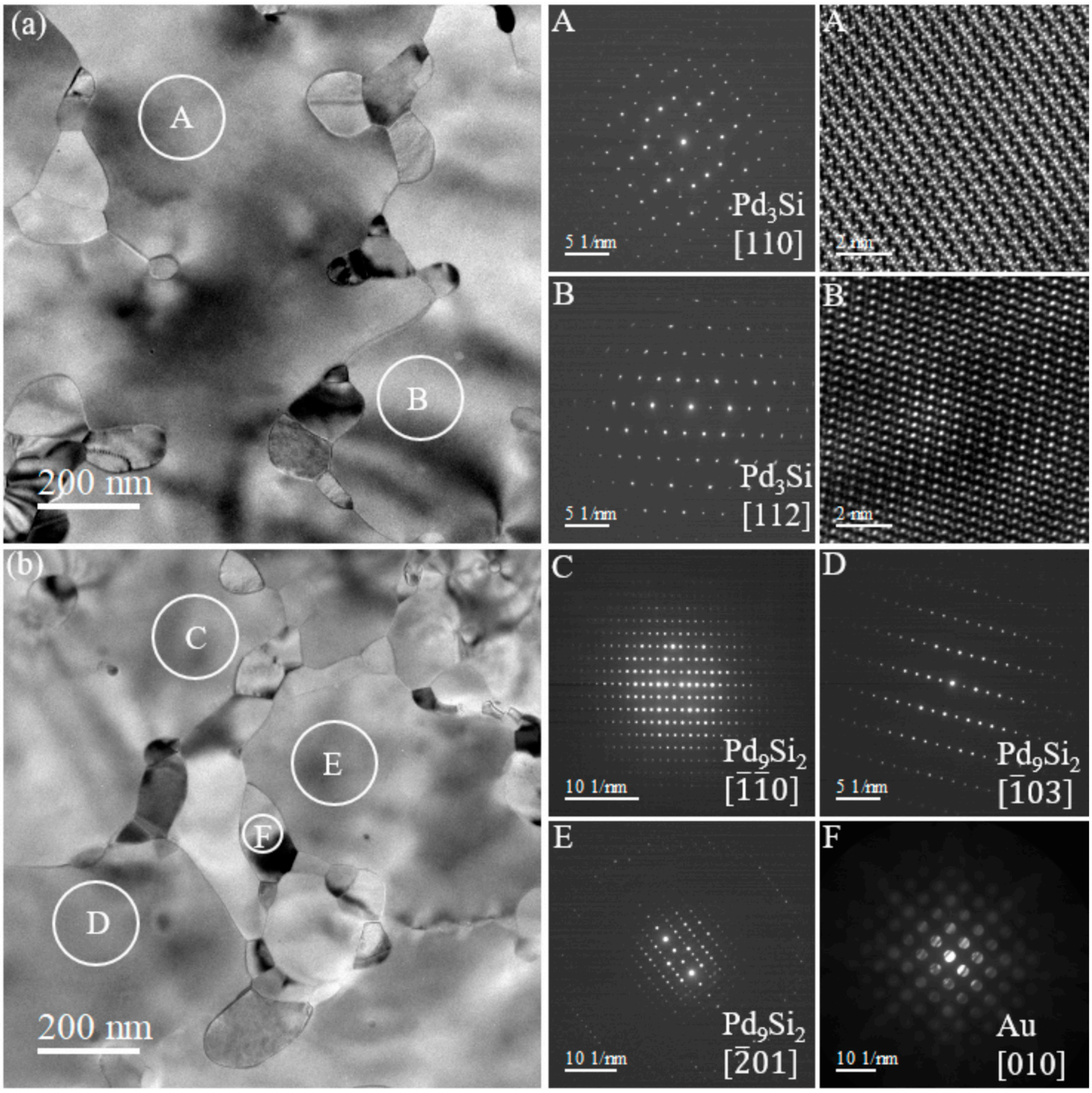

Figure S1. The final products of heating treatment of $Pd_{78}Au_4Si_{18}$ thin film at around 900 K. (a) Pd_3Si intermetallic phase and the corresponding SAED patterns and HRTEM lattice images of the regions marked by A and B. (b) Pd_9Si_2 intermetallic phase and the SAED patterns and HRTEM lattice images of the regions marked by C, D, E, and F.

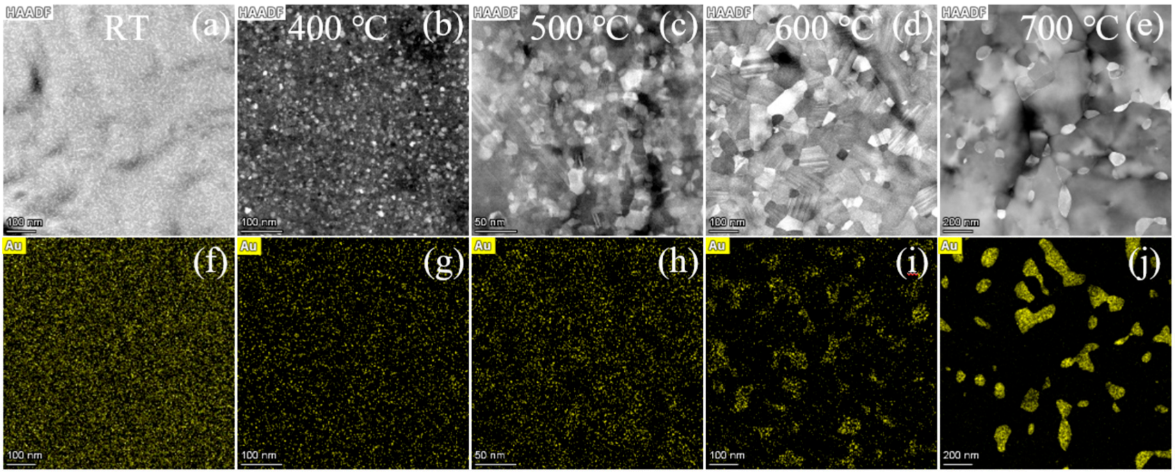

Figure S2. HAADF-STEM images (a–e) and EDXS mappings (f–j) of the $Pd_{78}Au_4Si_{18}$ thin film at different temperatures.
